# Association between DNA methylation in cord blood and maternal smoking: The Hokkaido Study on Environment and Children’s Health

**DOI:** 10.1038/s41598-018-23772-x

**Published:** 2018-04-04

**Authors:** Kunio Miyake, Akio Kawaguchi, Ryu Miura, Sachiko Kobayashi, Nguyen Quoc Vuong Tran, Sumitaka Kobayashi, Chihiro Miyashita, Atsuko Araki, Takeo Kubota, Zentaro Yamagata, Reiko Kishi

**Affiliations:** 1 Department of Health Sciences, Graduate School of Interdisciplinary Research, University of Yamanashi, Yamanashi, Japan; 2 Yamanashi Community Medicine Support Center, University of Yamanashi Hospital, Yamanashi, Japan; 3 Center for Environmental and Health Sciences, Hokkaido University, Hokkaido, Japan; 4 Faculty of Child Studies, Seitoku University, Iwase 550, Chiba, Japan

## Abstract

Maternal smoking is reported to cause adverse effects on the health of the unborn child, the underlying mechanism for which is thought to involve alterations in DNA methylation. We examined the effects of maternal smoking on DNA methylation in cord blood, in 247 mother–infant pairs in the Sapporo cohort of the Hokkaido Study, using the Infinium HumanMethylation 450K BeadChip. We first identified differentially methylated CpG sites with a false discovery rate (FDR) of <0.05 and the magnitude of DNA methylation changes (|β| >0.02) from the pairwise comparisons of never-smokers (Ne-S), sustained-smokers (Su-S), and stopped-smokers (St-S). Subsequently, secondary comparisons between St-S and Su-S revealed nine common sites that mapped to *ACSM3*, *AHRR*, *CYP1A1*, *GFI1*, *SHANK2*, *TRIM36*, and the intergenic region between *ANKRD9* and *RCOR1* in Ne-S vs. Su-S, and one common CpG site mapping to *EVC2* in Ne-S vs. St-S. Further, we verified these CpG sites and examined neighbouring sites using bisulfite next-generation sequencing, except for *AHRR* cg21161138. These changes in DNA methylation implicate the effect of smoking cessation. Our findings add to the current knowledge of the association between DNA methylation and maternal smoking and suggest future studies for clarifying this relationship in disease development.

## Introduction

The concept of Developmental Origins of Health and Disease (DOHaD) suggests that unfavourable changes in the environment during prenatal and early postnatal periods give rise to health issues later in life, emphasising the importance of environmental exposure during early development. In light of this notion, the mother’s lifestyle, particularly smoking, and its adverse effects on the child’s health have drawn considerable attention. Exposure to tobacco smoke during prenatal period has been found to be directly related to low birth weight and small foetal size^1^, increased risk of perinatal death^2^, and many health problems, including asthma, cardiovascular disease, and poor mental health^3^, via unknown mechanisms. Despite the increasing concerns of smoking on child health, approximately 13% of women in Northern Japan are reported to smoke during pregnancy^4^. The numbers are similar in USA (12.3%)^5^ and Europe^6^, and although this trend is starting to decline, 5%–10% of women still smoke during pregnancy in Scandinavian countries^7^.

Epigenetics regulates gene expression without altering the DNA sequence and is suggested as a key mechanism underlying the effect of environmental factors on neonatal health. Epigenetics comprises three major mechanisms: DNA methylation, histone modifications, and RNA-based regulations. DNA methylation has been shown to play critical roles in development^8^. Previous studies have detected alterations in both global DNA methylation and CpG site methylation in different tissues and cell types between smokers and non-smokers. Global hypomethylation has been detected in buccal cells and peripheral blood granulocytes of children exposed to prenatal smoking^9,10^, but no changes have been observed in the first trimester^11^. Later, more specific loci and genes related to maternal smoking during pregnancy have been revealed using the Infinium HumanMethylation27 BeadChip^12^ or the Infinium HumanMethylation 450 K BeadChip (HM450K)^13–18^, which covers >27,000 and 450,000 methylation sites across the human genome, respectively. Multiple studies using the HM450K assay have shown five genes to be associated with maternal smoking, including Aryl-Hydrocarbon Receptor Repressor (*AHRR*), Cytochrome P450 Family 1 Subfamily A Member 1 (*CYP1A1*), Growth Factor Independent 1 Transcriptional Repressor (*GFI1*), Myosin IG (*MYO1G*), and Contactin Associated Protein Like 2 (*CNTNAP2*)^13,14,16,17^.

Importantly, changes in DNA methylation patterns of some genes, including *AHRR*, *MYO1G*, *CYP1A1* and *CNTNAP2*, that occurred in newborns were sustained until childhood or adulthood, whereas those in other genes, such as *GFI1*, showed a trend of recovery but did not reach the level of the unexposed group^15,17–20^. Recently, it was shown that alterations in DNA methylation patterns as a result of prenatal tobacco smoke exposure were sustained until the third or fourth decade of life^21^. These findings demonstrate the long-term effect of prenatal tobacco smoke exposure on DNA methylation, supporting the DOHaD concept.

It should be noted that the HM450K analysis mentioned above were conducted in European or American populations. Although no samples from Asian populations were included, a previous study showed a high divergence in DNA methylation patterns between populations^22^. Specifically, 32% of the observed sites varied between populations of European origin and African origin; more than 50% of the differentiated sites had a methylation disparity >5%. To our knowledge, there have been neither DNA methylation-based studies nor epigenome-wide association studies (EWAS) have focused on prenatal tobacco smoke exposure in Asian population. Thus, we tested cord blood samples obtained from 247 mother–infant pairs in the Sapporo cohort using HM450K analysis. The objectives of this study were three-fold: (1) to replicate the findings of previous studies on a Japanese population, (2) to gain further insights into the changes in DNA methylation patterns due to smoking cessation during pregnancy and (3) to validate the results at a higher resolution using bisulfite sequencing.

## Results

### Epigenome-wide analysis of DNA methylation and maternal smoking during pregnancy

Characteristic features of 291 mother–infant pairs are shown in Table 1. No differences were observed among the mothers across the different mother–infant pairs for their maternal age, BMI before pregnancy and parity. Additionally, mothers who never smoked tended to have higher education and were less likely to have been exposed to passive smoking at work place or to have sustained smoking partners. Moreover, there were no differences between sex, gestational age and birth weight of the infants across the different mother–infant pairs.

**Table 1:** Characteristics of study population.

	Maternal smoking status						
	**All (n = 291)**	**Never-smokers (n = 124)**	**Stopped smokers before pregnancy (n = 44)**	**Stopped smokers after confirming pregnancy (n = 77)**	**Sustained smokers during pregnancy (n = 46)**	**P value (among 4 groups)**	**P-value (Never-smokers vs sustained-smokers)**
Mothers							
Maternal Age (years)	30.1 ± 5.0	30.7 ± 4.9	31.1 ± 4.0	29.0 ± 5.0	29.1 ± 5.5	0.033*	0.076
Maternal BMI before pregnancy (kg/m^2^)	20.9 ± 2.9	20.8 ± 2.9	20.7 ± 1.6	21.1 ± 3.3	20.9 ± 3.2	0.835	0.865
Parity							
0	150 (51.5)	66 (53.2)	15 (34.1)	47 (61.0)	22 (47.8)	0.036*	0.531
≥1	141 (48.5)	56 (46.8)	29 (65.9)	30 (39.0)	24 (52.2)		
Maternal Educational level (years)							
≤12	131 (45.0)	54 (36.3)	20 (45.5)	37 (48.1)	29 (63.0)	0.017*	0.002**
>12	160 (55.0)	79 (63.7)	24 (54.5)	40 (51.9)	17 (37.0)		
Partner’s smoking status							
Mothers with partners of never smokers	44 (15.1)	27 (21.8)	8 (18.2)	7 (9.1)	2 (4.3)	0.001**	0.003**
Mothers with partners of stopped smoking before pregnancy	35 (12.0)	21 (16.9)	8 (18.2)	3 (3.9)	3 (6.5)		
Mothers with partners of stopped smoking after confirming pregnancy	4 (1.4)	3 (2.4)	0 (0.0)	1 (1.3)	0 (0.0)		
Mothers with partners of sustained smoking during pregnancy	207 (71.1)	73 (58.9)	28 (63.6)	66 (85.7)	40 (87.0)		
Unknown	1 (0.3)	0 (0.0)	0 (0.0)	0 (0.0)	1 (2.2)		
Infants							
Sex							
Male	131 (45.0)	52 (41.9)	27 (61.4)	36 (46.8)	16 (34.8)	0.064	0.398
Female	160 (55.0)	72 (58.1)	17 (38.6)	41 (53.2)	30 (65.2)		
Gestational age (weeks)	39.4 ± 1.0	39.3 ± 1.1	39.3 ± 1.2	39.5 ± 0.9	39.5 ± 1.0	0.225	0.221
Birth weight (g)	3129.2 ± 332.7	3111.3 ± 324.8	3099.9 ± 382.3	3176.8 ± 339.3	3130.2 ± 292.2	0.519	0.729

n (%) or mean ± standard deviation. ANOVA, independent t-test and chi-squared test.

*P < 0.05; **P < 0.01; ***P < 0.001.

**Figure 1:**
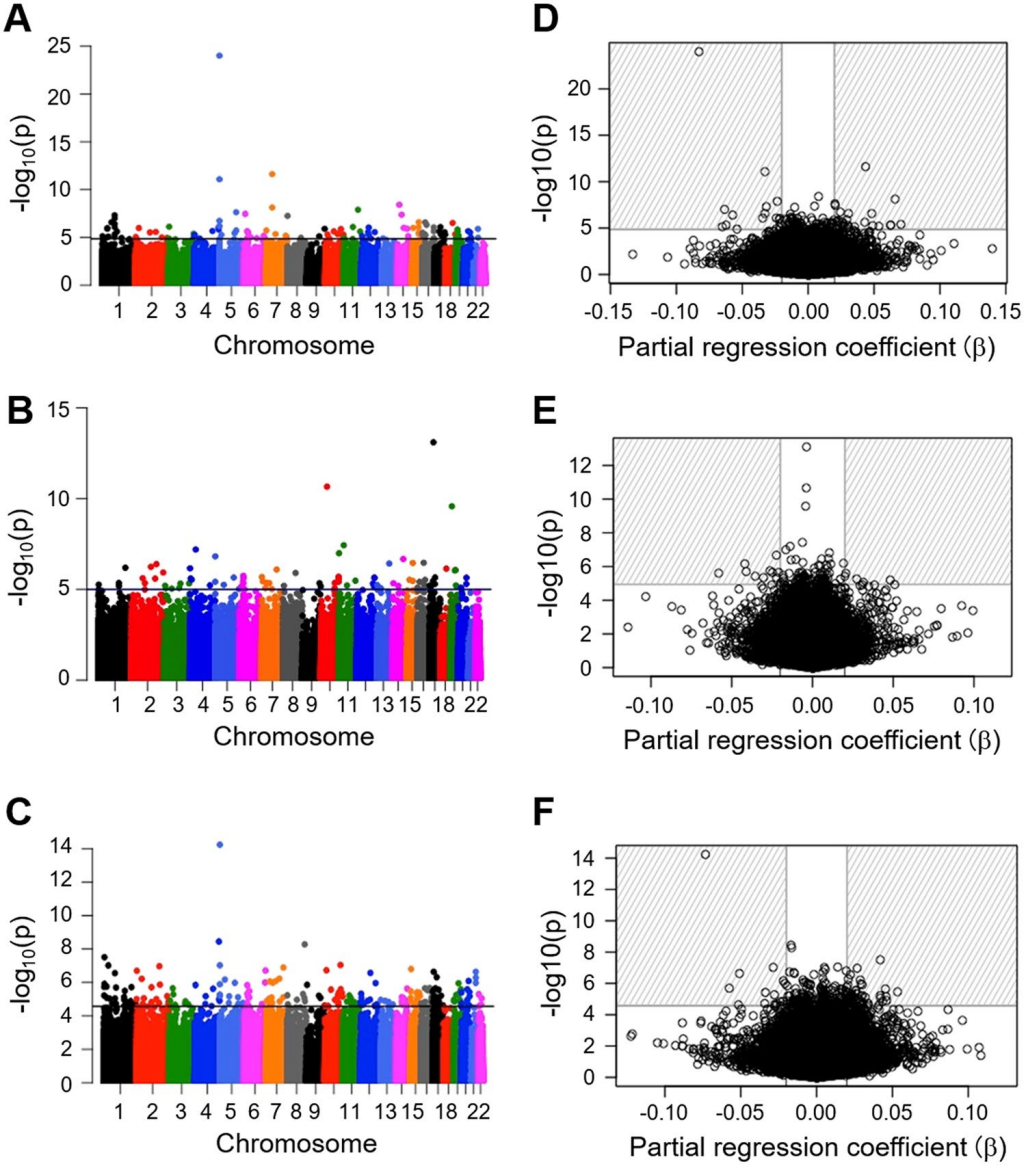
Epigenome-wide association between the smoking status of pregnant mothers and the methylation status of 426,576 CpG sites in cord blood samples. Manhattan plots (left panel) and volcano plots (right panel) showing primary comparisons between never-smokers (Ne-S) vs. sustained-smokers (Su-S) (**A** and **D**), stopped-smokers (St-S) vs. Su-S (**B** and **E**) and Ne-S vs. St-S (**C** and **F**). CpG sites with FDR <0.05 are indicated above the horizontal line in Manhattan plots; different colours represent different chromosomes. CpG sites with FDR >0.5 and |β| >0.2 are located in the striped region in volcano plots.

Among the 291 mother–infant pairs, 44 mothers had smoked at some point during their life but had stopped smoking before their pregnancy. These 44 mother–infant pairs were excluded from the study, as the duration of their non-smoking periods was unknown. The remaining 247 mother–infant pairs were categorised into three exposure categories: 124 never-smokers (Ne-S), 46 sustained-smokers (Su-S) and 77 stopped-smokers (St-S) who stopped smoking after the confirmation of their pregnancy. To investigate the effect of smoking on DNA methylation patterns, differentially methylated (DM) sites between the children of different groups, i.e. Ne-S vs. Su-S, Ne-S vs. St-S and St-S vs. Su-S were identified and −log 10 *p*-values of each comparison were plotted (Fig. 1A–C). Data revealed 121, 46 and 99 DM sites in Ne-S vs. Su-S, Ne-S vs. St-S and St-S vs. Su-S comparisons, respectively, with a false discovery rate (FDR) of <0.05 (indicated by data points above the horizontal line in Fig. 1A–C).

Next, we inspected DNA methylation change using partial regression coefficient β. Only the DM CpG sites with |β| >0.02 were chosen. Of the 121 DM sites with significant FDR identified in the Ne-S vs. Su-S comparison, 46 met the criteria of β-value and mapped to 27 genes (Fig. 1D and Supplementary Table S1). In the Ne-S vs. St-S comparison, only 15 CpG sites fitted the FDR and β-value requirements (Fig. 1E and Supplementary Table S2), whereas the comparison between St-S vs. Su-S resulted in 64 DM sites fitting the FDR and β-value requirements (Fig. 1F and Supplementary Table S3).

### Overlapping differentiated DNA methylation sites in never-smoking and stop-smoking groups compared with smoking group

**Figure 2:**
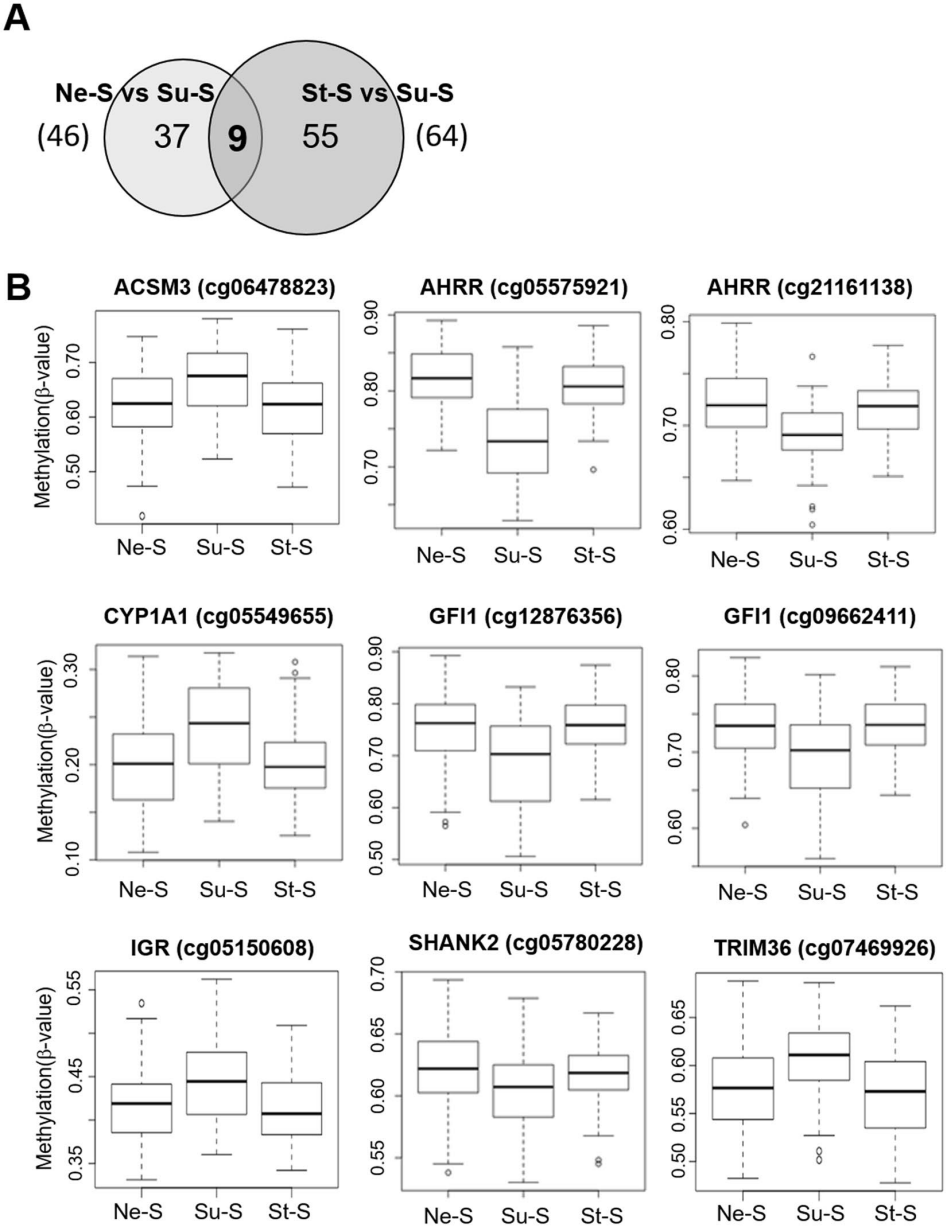
Secondary comparison of differentially methylated CpG sites identified in the Ne-S vs. Su-S comparison relative to those identified in the St-S vs. Su-S comparison. (**A**) Venn diagram showing 9 CpG sites that were common between the Ne-S vs. Su-S comparison (46 sites) and the St-S vs. Su-S comparison (64 sites). (**B**) Box plots showing methylation rates (β-value) at 9 common CpG sites.

Taking into account that smoking cessation during pregnancy may prevent adverse effects of smoking on the child’s health, we aimed to identify DM sites associated with maternal smoking that were not altered if the mother quit smoking. To this effect, we compared DM sites identified in the Ne-S vs. Su-S comparison to those identified in the St-S vs. Su-S comparison to identify the DM sites that were common to both comparisons. These DM sites would potentially represent CpG sites that were not affected, if the mother stopped smoking early in the pregnancy. Comparison between 46 Ne-S vs. Su-S DM sites and 64 St-S vs. Su-S DM sites revealed 9 common CpG sites (Fig. 2A). Of these nine CpG sites, eight mapped to six genes, including Acyl-CoA Synthetase Medium Chain Family Member 3 (*ACSM3*) (cg06478823), *AHRR* (cg05575921 and cg21161138), *CYP1A1* (cg05549655), *GFI1* (cg12876356 and cg09662411), SH3 And Multiple Ankyrin Repeat Domains 2 (*SHANK2*) (cg05780228) and Tripartite Motif Containing 36 (*TRIM36*) (cg07469926) and one mapped to the intergenic region (IGR) between Ankyrin Repeat Domain 9 (*ANKRD9*) and REST Corepressor 1 (*RCOR1*) (cg05150608) (Fig. 2B and Table 2). All nine CpG sites showed similar methylation rates (β-value) in Ne-S and St-S groups, except for a significant difference in cg05575921 of *AHRR*. Four CpG sites in *ACSM3*, *CYP1A1*, *TRIM36* and the IGR were found to be hypermethylated, whereas five CpG sites in *AHRR*, *GFI1* and *SHANK2* were found to be hypomethylated in the Su-S group (Fig. 2B and Table 2).

**Table 2:** Details for common CpG sites from secondary comparisons.

**CpG site**	**Chr**.^**a**^	**Gene region**	**Ne-S** ^**b**^ **vs Su-S** ^**c**^			**Ne-S vs St-S** ^**d**^			**St-S vs Su-S**			**Mean (SD** ^**e**^ **) methylation (ratio)**		
			**P-value**	**FDR** ^**f**^	**Beta** ^**g**^	**P-value**	**FDR**	**Beta**	**P-value**	**FDR**	**Beta**	**Ne-S**	**St-S**	**Su-S**
cg06478823	16	ACSM3	2.69E-07	7.18E-03	0.048	0.791	0.974	0.002	2.40E-05	0.048	0.041	0.724 (0.059)	0.721 (0.064)	0.766 (0.064)
cg05575921	5	AHRR	9.65E-25	4.12E-19	−0.083	0.032	0.528	−0.010	5.81E-15	2.48E-09	−0.073	0.819 (0.040)	0.805 (0.038)	0.737 (0.054)
cg21161138	5	AHRR	8.22E-12	1.17E-06	−0.033	0.638	0.946	−0.002	9.50E-08	0.006	−0.029	0.721 (0.032)	0.717 (0.026)	0.690 (0.031)
cg05549655	15	CYP1A1	2.63E-07	7.18E-03	0.031	0.220	0.796	0.006	9.95E-06	0.036	0.028	0.201 (0.045)	0.200 (0.040)	0.242 (0.048)
cg12876356	1	GFI1	9.67E-08	3.44E-03	−0.063	0.913	0.990	0.001	2.03E-06	0.021	−0.058	0.752 (0.066)	0.754 (0.057)	0.688 (0.087)
cg09662411	1	GFI1	8.19E-07	0.014	−0.035	0.451	0.899	0.004	1.91E-05	0.044	−0.034	0.730 (0.041)	0.734 (0.039)	0.694 (0.059)
cg05150608	14	IGR	7.49E-06	0.036	0.030	0.945	0.994	0.000	2.56E-05	0.048	0.034	0.417 (0.040)	0.414 (0.042)	0.448 (0.053)
cg05780228	11	SHANK2	8.23E-07	0.014	−0.024	0.493	0.912	−0.002	1.38E-05	0.038	−0.022	0.622 (0.030)	0.618 (0.025)	0.601 (0.034)
cg07469926	5	TRIM36	3.64E-06	0.025	0.027	0.424	0.890	−0.004	1.06E-06	0.016	0.029	0.578 (0.044)	0.570 (0.043)	0.605 (0.045)
cg01290904	4	EVC2	5.90E-06	0.031	0.023	2.52E-06	0.031	0.021	3.09E-01	0.799981	0.005709	0.581 (0.042)	0.593 (0.040)	0.601 (0.041)

^a^Chromosome. ^b^Never-smoker. ^c^Sustained-smoker. ^d^Stopped-smoker. ^e^Standard deviation. ^f^False discovery rate. ^g^Partial regression coefficient for the magnitude of the effect on DNA methylation change

Above the line: common CpG sites from comparison between (Ne-S vs Su-S) and (St-S vs Su-S).

Below the line: common CpG sites from comparison between (Ne-S vs Su-S) and (Ne-S vs St-S).

**Figure 3:**
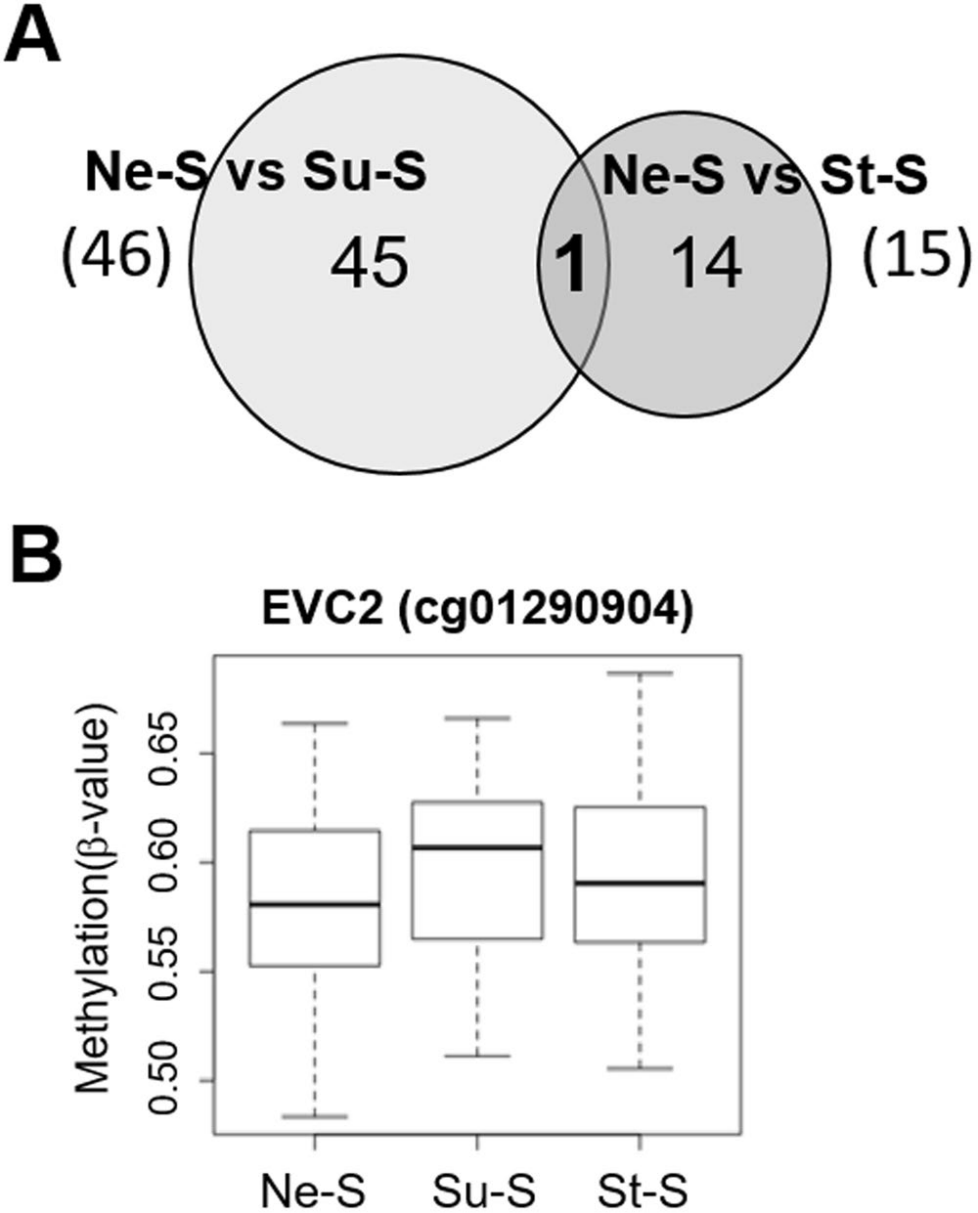
Secondary comparison of differentially methylated CpG sites identified in the Ne-S vs. Su-S comparison relative to those identified in the Ne-S vs. St-S comparison. (**A**) Venn diagram showing one CpG site that was common between the Ne-S vs. Su-S comparison (46 sites) and the Ne-S vs. St-S comparison (15 sites). (**B**) Box plots showing methylation rates (β-value) at one common CpG site.

On the other hand, to identify the CpG sites whose methylation pattern was altered even after the mother stopped smoking, we compared the 46 CpG sites identified in the Ne-S vs. Su-S with the 15 sites identified in the Ne-S vs. St-S comparison. Data revealed only one common CpG site (cg01290904). This CpG site mapped to the EvC Ciliary Complex Subunit 2 (*EVC2*) gene and was hypermethylated in both the Su-S and St-S groups compared with the Ne-S group (Fig. 3 and Table 2).

### Verification of DNA methylation analysis using bisulfite sequencing

Despite being widely used in the field of epigenetics for large-scale DNA methylation profiling, HM450K and analysis of tandem HM450K have their limitations, e.g., restriction to predesigned CpG sites or complications in data normalisation and analysis^23^. Moreover, some of the selected CpG sites from our secondary comparison fall within the list of suspected low-quality probes provided by Naeem *et al*.^24^, namely *ACSM3* cg06478823, *AHRR* cg21161138, and *SHANK2* cg05780228 (Supplementary Tables S1, S2, and S3). Thus, we further verified our findings using next-generation sequencing (NGS) technique following bisulfite conversion. Because of their locations in GC-rich regions, suitable primers could not be designed for cg12876356 and cg09662411 located in *GFI1*, Therefore, *GFI1* was excluded from verification experiments.

**Figure 4:**
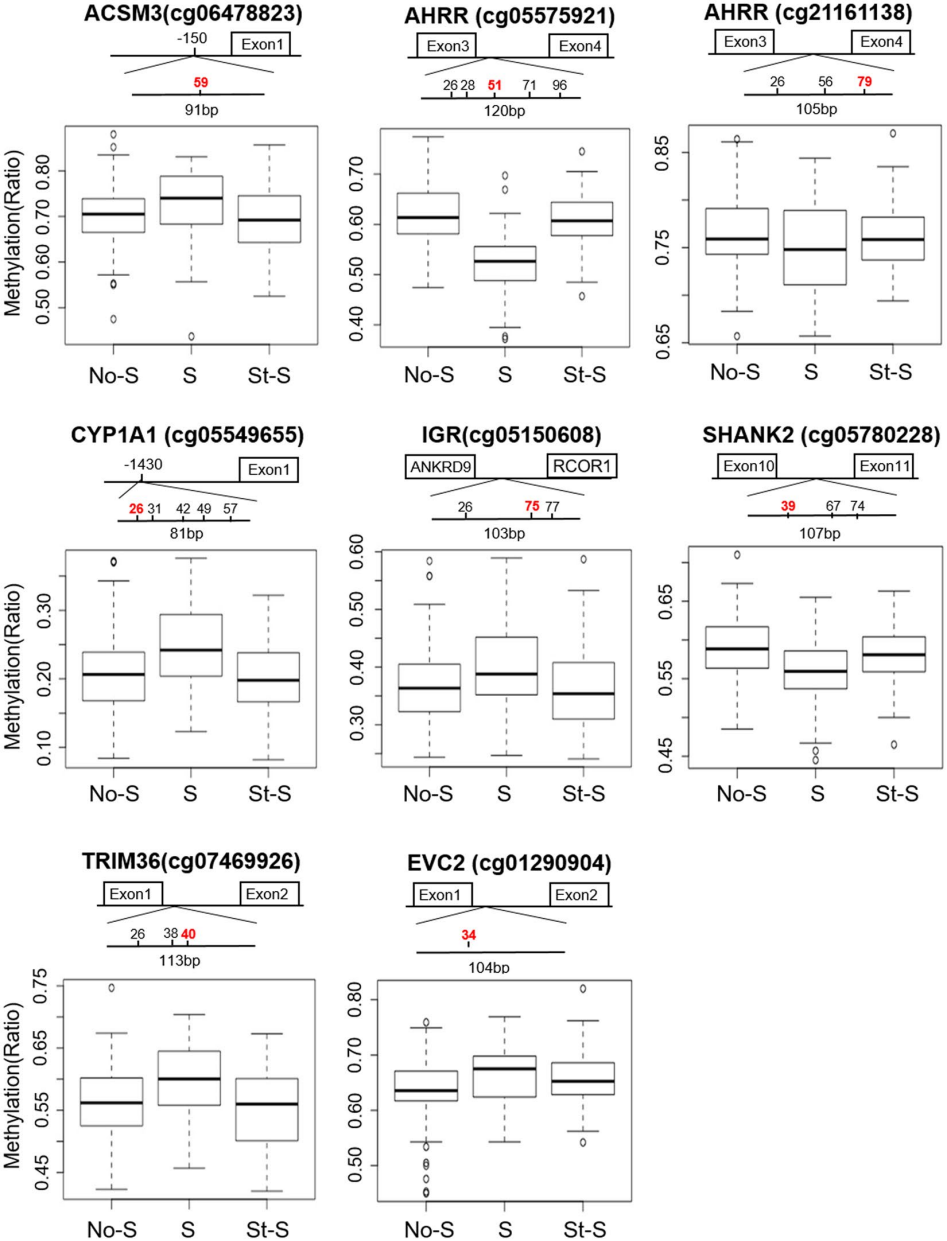
Next-generation sequencing (NGS) analysis of eight CpG sites from secondary comparisons after excluding outliners in AHRR cg21161138, SHANK2 cg05780228, EVC2 cg01290904, and 2 sites associated with *GFI1* due to technical issue. The upper part of each panel represents the length and schematic position in each sequence. Numbers represent the CpG sites; the same sites in the HumanMethylation450K array (HM450K) are marked in red. DNA methylation ratios from NGS analysis of the same CpG sites in HM450K are shown in box plots in the lower part of each panel.

**Figure 5:**
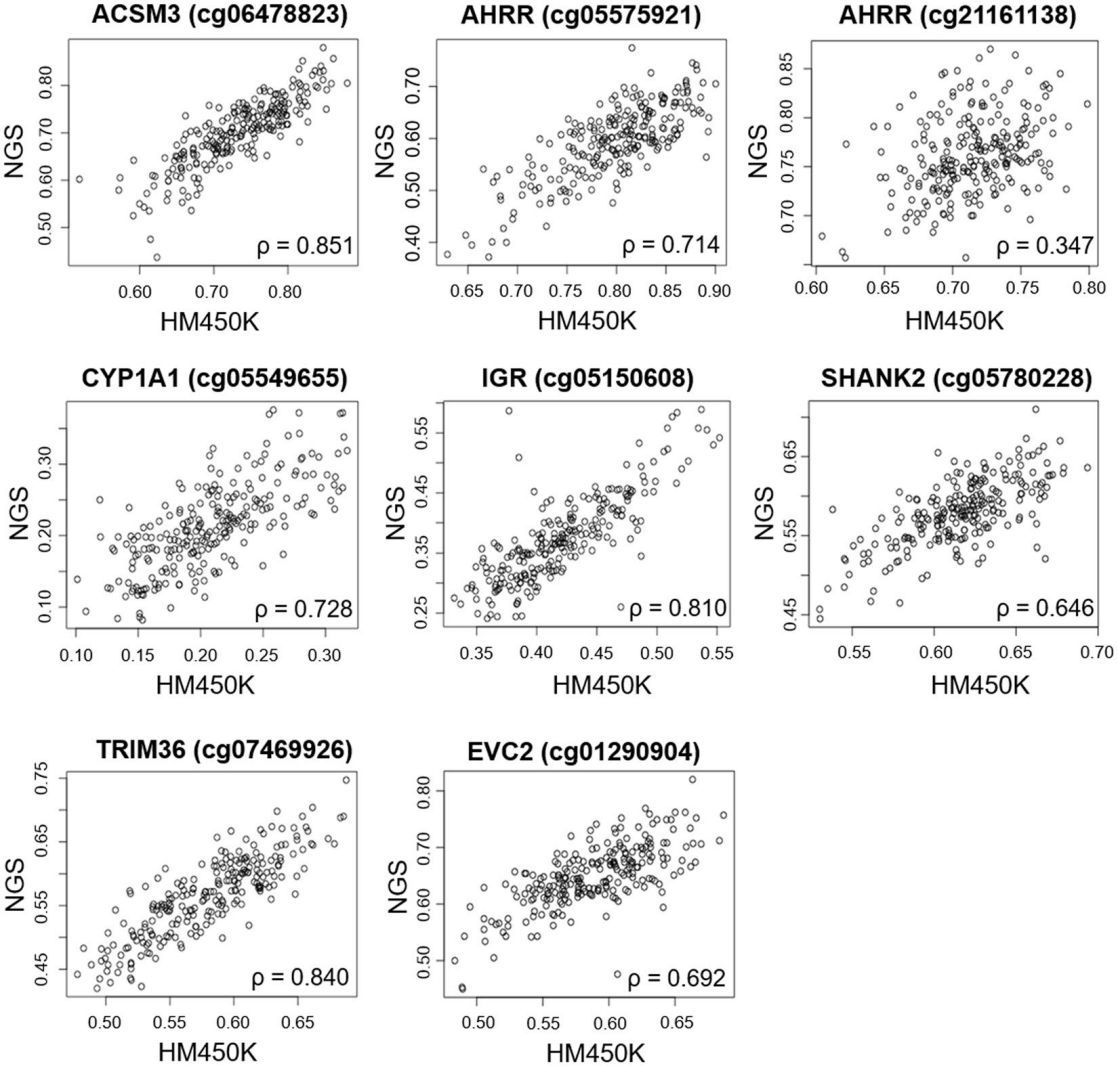
Correlation between HumanMethylation450K array (HM450K) and next generation sequencing (NGS) data analysis for the DNA methylation status of eight CpG sites after removing outliers. Values of Spearmen correlation coefficient (ρ) are indicated.

Due to some outliers in the NGS result for *AHRR* cg21161138, *SHANK2* cg05780228, and *EVC2* cg01290904 (black arrows in Supplementary Fig. S1), we chose to perform analysis both including and excluding outliers. Our NGS analysis showed that seven out of eight CpG sites exhibited similar DNA methylation profiles to that of HM450K, with the exception of cg21161138 within the *AHRR* gene, which showed no changes in methylation rates between the Ne-S, Su-S and St-S groups (Fig. 4, bottom panel in each target, Table 3, and Supplementary Fig. S1). The statistical significance in NGS analysis remained the same for all sites after discounting outliers, except for cg012090904 within the *EVC2* gene, which fell slightly outside the range of significance (p = 0.0526) after discounting outliers (Table 3). Correlation analysis revealed a moderate to strong positive correlation (ρ > 0.6) between DNA methylation ratio at the seven CpG sites determined by HM450K analysis and by NGS analysis, excluding *AHRR* cg21161138 (ρ = 0.347 and ρ = 0.331 after and before discounting outlier, respectively) (Fig. 5 and Supplementary Fig. S2).

**Table 3:** Verification result for selected CpG sites using Next Generation Sequencing.

**Gene region**	**CpG site** ^**a**^	**P-value**			**Mean (SD** ^**e**^ **) methylation (ratio)**		
		**Ne-S** ^**b**^ **vs Su-S** ^**c**^	**Ne-S vs St-S** ^**d**^	**St-S vs Su-S**	**Ne-S**	**St-S**	**Su-S**
ACSM3	cg59 (cg06478823)	0.032*	0.728	0.033*	0.700 (0.062)	0.690 (0.078)	0.722 (0.080)
AHRR	cg26	1.17E-10**	0.258	1.55E-08**	0.644 (0.052)	0.636 (0.049)	0.564 (0.068)
	cg28	6.64E-12**	0.205	6.24E-09**	0.640 (0.054)	0.630 (0.050)	0.549 (0.067)
	cg51 (cg05575921)	1.05E-11**	0.625	9.76E-11**	0.615 (0.060)	0.609 (0.050)	0.519 (0.072)
	cg71	3.73E-12**	0.587	1.48E-10**	0.738 (0.064)	0.733 (0.057)	0.611 (0.097)
	cg96	1.33E-10**	0.388	6.18E-08**	0.679 (0.058)	0.670 (0.057)	0.579 (0.082)
AHRR	cg26	0.964 (0.984)	0.768 (0.826)	0.980 (0.980)	0.854 (0.034)	0.851 (0.037)	0.854 (0.035)
	cg56	0.007**(0.656)	0.827 (0.892)	0.042* (0.429)	0.785 (0.150)	0.814 (0.111)	0.768 (0.150)
	cg79 (cg21161138)	0.064 (0.064)	0.669 (0.576)	0.280 (0.350)	0.767 (0.040)	0.755 (0.063)	0.749 (0.046)
CYP1A1	cg26 (cg05549655)	8.47E-04**	0.941	7.06E-04**	0.210 (0.061)	0.202 (0.053)	0.245 (0.061)
	cg31	0.008**	0.986	0.005**	0.242 (0.066)	0.236 (0.054)	0.274 (0.060)
	cg42	0.009**	0.312	3.67E-04**	0.480 (0.081)	0.461 (0.073)	0.518 (0.071)
	cg49	0.166	0.288	0.007**	0.470 (0.070)	0.453 (0.065)	0.491 (0.070)
	cg57	8.93E-4** (0.002**)	0.781 (0.781)	7.07E-5** (2.19E-04**)	0.566 (0.077)	0.558 (0.064)	0.604 (0.069)
IGR	cg26	0.72 (0.849)	0.600 (0.600)	0.303 (0.422)	0.371 (0.076)	0.367 (0.070)	0.374 (0.087)
	cg75 (cg05150608)	0.024*	0.736	0.010*	0.368 (0.066)	0.365 (0.071)	0.403 (0.080)
	cg77	0.044*	0.707	0.041*	0.376 (0.078)	0.372 (0.085)	0.415 (0.110)
SHANK2	cg39 (cg05780228)	2.50E-04** (1.98E-04**)	0.287 (0.246)	0.034* (0.034*)	0.590 (0.042)	0.579 (0.040)	0.558 (0.046)
	cg67	0.09 (0.120)	0.986 (0.964)	0.163 (0.163)	0.937 (0.018)	0.938 (0.013)	0.932 (0.017)
	cg74	0.025*	0.793	0.022*	0.848 (0.027)	0.849 (0.028)	0.837 (0.027)
TRIM36	cg26	0.003**	0.777	0.003**	0.549 (0.085)	0.538 (0.088)	0.602 (0.098)
	cg38	0.031*	0.562	0.009**	0.569 (0.082)	0.556 (0.087)	0.606 (0.088)
	cg40 (cg07469926)	0.005**	0.678	0.002**	0.563 (0.057)	0.551 (0.065)	0.595 (0.063)
EVC2	cg34 (cg01290904)	0.007** (0.007)**	0.0526 (0.042*)	0.429 (0.527)	0.637 (0.056)	0.658 (0.088)	0.667 (0.054)

^a^CpG sites are numbered according to their position on the sequencing products, overlapped CpG sites with Human Methylation 450K array are indicated in parentheses. ^b^Never-smoker. ^c^Sustained-smoker. ^d^Stopped-smoker. ^e^Standard deviation.

Steel-Dwass tests. *P < 0.05; **P < 0.01, P value from analysis before removal of outliers are indicated in parentheses.

Because NGS provides single base-resolution, we were able to examine adjacent CpG sites falling within the region spanned by the primers (Fig. 4, upper panel in each target). Of the 16 neighbouring CpG sites, methylation patterns of 13 sites were consistent with those determined using HM450K for Su-S and St-S groups (Table 3 and Supplementary Fig. S3). Notably, multiple CpG sites near *AHRR* cg05575921, *CYP1A1* cg05549655 and *TRIM36* cg07469926 were altered by smoking and were not changed if smoking was stopped in early pregnancy (Table 3 and Supplementary Fig. S3A,S3C and S3F), suggesting that these regions are highly susceptible to smoking.

## Discussion

In the current study, we conducted an EWAS using HM450K analysis in a Japanese population from the Sapporo cohort of the Hokkaido Study to address the current interest in DNA methylation alteration due to prenatal tobacco smoke exposure. Considering the beneficial effects of quitting smoking early in the pregnancy, we identified nine CpG sites located in six genes and an intergenic region, whose DNA methylation patterns were altered by smoking but recovered after early smoking cessation. However, the methylation pattern of CpG sites in *EVC2* (cg01290904) was observed even after the cessation of smoking. We authenticated the findings of HM450K analysis using bisulfite sequencing, with the exclusion of CpG sites in *GFI1* due to technical issues. We found a relatively strong correlation between the two approaches for the methylation status of CpG sites, except for the sites in *AHRR* (cg21161138).

In a primary comparison between each smoking group, CpG sites that passed both criteria, including epigenome-wide significance level (FDR <0.05) and effect size (|β| >0.02), were selected. We chose a difference of 2% to narrow down the CpG sites based on a previous publication, which defined the mean methylation change for both hyper- and hypomethylation as 2%^16^. Moreover, a recent review summarised that most environmental exposure studies have reported changes in DNA methylation from 2–10% and called for focusing on small magnitude alterations^25^. In the EWAS comparison between Ne-S and Su-S groups, we replicated the CpG sites that were consistent with previous studies, particularly the hypomethylated sites in *AHRR* and *GFI1* and the hypermethylated sites in *CYP1A1* and *MYO1G*^13,14,16,17^. Among the 64 DM CpG sites, cg05575921 associated with *AHRR* was found to be consistently replicated across studies assessing DNA methylation in cord blood or newborn blood^6,13,14,17^. In detail, all CpG sites identified in *GFI1*, *CYP1A1* and *MYO1G* in this study were also reported in previous studies; *AHRR* cg21161138 was reported by Joubert *et al*.^13^, Kupers *et al*.^14^ and Markunas *et al*.^16^ but not by Richmond *et al*.^17^. Another *AHRR*-associated CpG site, cg17924476, although not reported in studies using cord blood or newborn blood, was detected in adolescents exposed to maternal smoking^15^. Moreover, multiple DM CpG sites, including two in *MARCH3*, two in *FLYWCH1* and three in *ACSM3* were not identified in previous studies, which suggests these to be novel targets affected by prenatal tobacco smoke exposure and warrants further studies for validation. We did not detect any variation consistent with the genome-wide significant criteria in *CTCNAP2* cg25949550, a CpG site that was agreeable with previous studies (data not shown). This may be explained by the differences between populations, as our study was performed in an Asian population when others were conducted mostly in Caucasian populations. Moreover, evidence suggests that genetic background and genetic variants effect differences in DNA methylation at CpG sites associated with maternal smoking^19,26,27^. Although we did not investigate genetic variants in our study, we attempted to search for single-nucleotide polymorphism (SNP) sites in *CTCNAP2* and several novel genes that may affect the reported CpG sites from the Infinium HD Methylation SNP List provided by Illumina. We then compared SNP sites between Japanese and Caucasian populations using the 1000 Genomes Browser (https://www.ncbi.nlm.nih.gov/variation/tools/1000genomes/). However, we detected a higher frequency of SNP sites that may affect the methylation rate at cg05150608 only in the intergenic region between *ANKRD9* and *RCOR1* (Supplementary Table S4). Nevertheless, a study comparing European and African populations found that genetic variances may explain as much as half of the diversity in DNA methylation between populations; the remaining variance is thought to be due to complex gene-gene and gene-environment interactions^22^.

Considering that the negative effects of prenatal tobacco smoke exposure can be diminished if the mother quits smoking in the first trimester of pregnancy, it is vital to determine the critical window of exposure. We conducted a secondary comparison to identify CpG sites that overlapped across the different primary comparisons, with the aim to focus on CpG sites that may be specific to the St-S group. Five of the nine CpG sites identified for unaltered DNA methylation patterns if the mothers stopped smoking early during pregnancy, namely *AHRR* cg05575921, *AHRR* cg21161138, *CYP1A1* cg05549655, *GFI1* cg12876356 and *GFI1* cg09662411 were previously reported, as discussed above. Except for *AHRR* cg21161138, which showed a weak correlation with HM450K and no significant difference in methylation between groups in NGS, we successfully verified the changes in DNA methylation at *AHRR* cg05575921 and *CYP1A1* cg05549655. Two CpG sites in *GFI1* gene were not verified by NGS due to technical issues. Novakovic *et al*. performed a detailed investigation on *AHRR* methylation and found no significant differences between children from women who never smoked and those who stopped smoking after confirmation of their pregnancy^19^. Similarly, Joubert *et al*. reported that alterations in DNA methylation occurred in the foetus only if women smoked past the 18^th^ week of pregnancy^28^. The authors further indicated that changes in DNA methylation patterns result from maternal smoking, and not from paternal smoking before pregnancy, or from an epigenetic inheritance of smoking effects from the grandmother to the mother. Richmond *et al*. found no differences at *AHRR* cg05575921 and *CYP1A1* cg05549655 between cord blood samples that were unexposed or exposed only during the first trimester^17^. The authors also reported the same finding in *GFI1*, although the CpG site identified in their study (cg09935388) was different from those identified in this study (cg12876356 and cg09662411). Our findings at *AHRR* cg05575921 were consistent with previously published data^19^, where hypomethylated state of CpG sites neighbouring cg05575921 was observed in the cord blood samples of mothers in the Su-S and Ne-S but not among those of mothers in the St-S. In adult smokers, stopping or reducing smoking was related to the recovery of DNA methylation status at *AHRR* in peripheral blood^29^. A very recent study examined *AHRR* and *CYP1A1* methylation and mRNA expression using first trimester foetal livers and placentas; significant changes in *AHRR* methylation were only observed in placentas from female foetus^30^. However, significant increases in the mRNA of *AHRR* in livers and of *CYP1A1* in placentas were detected, which suggests that smoking may have an early effect on gene expression before inducing noticeable alterations in DNA methylation^30^. Thus, the question regarding of the duration of maternal smoking exposure required to make remarkable and sustained changes in the DNA methylation level is still unclear and warrants further investigations. In total, together with other studies, our results further support *AHRR* as an exemplary gene for the interaction between environment and DNA methylation.

Amidst the reported genes associated with smoking, in particular maternal smoking, *AHRR* and *CYP1A1* were thoroughly investigated. Both *AHRR* and *CYP1A1* function downstream of AHR; while *AHRR* represses *AHR*, *CYP1A1* was activated by AHR^19^. Consistent with previous studies, we found a hypomethylated status of *AHRR* and a hypermethylated status of the *CYP1A1* promoter^13–19^. Moreover, it has been shown that the hypomethylation at *AHRR* cg05575921 is correlated with an increase in *AHRR* expression^31^. Notably, alterations in the DNA methylation status of both *AHRR* and *GFI1* in cord blood contributes to low birth weight of children of mothers in the Su-S group^14,32^. Specifically, hypomethylation cg05575921 was found to associate with low birth weight^33^. As discussed in Tian *et al*., AHRR cg05575921 hypomethylation was correlated with an increase in AHRR expression and several effects, including repression of oxygen transport-related embryonic globin genes, inhibition of cell growth and proliferation, and suppression of angiogenesis. Although genetic polymorphisms in *CYP1A1* have been implicated to affect birth weight and birth size^1^, the contribution of *CYP1A1* hypermethylation in cord blood has not been fully investigated. However, we did not observe significant change in birth weights between Ne-S, Su-S, and St-S. We can only assume that our relatively small sample size contributed to the absence of a low birth weight phenotype. In addition to being recognised as a risk factor for lower birth weight and birth size in newborns, intra utero tobacco smoke exposure has been shown to increase the risk of neurodevelopmental disorders, particularly autism spectrum disorder and attention deficit hyperactivity disorder (ADHD)^34^. This notion was recently addressed in a study investigating the association between DNA methylation in the peripheral blood of ADHD children and maternal smoking during pregnancy^32^. Despite the small size of the population studied, DNA methylation at *AHRR* and *GFI1*, but not at *CYP1A1*, were shown to be related to the severity of conduct disorder and ADHD in children of smoking mothers. Furthermore, maternal smoking has been shown to induce hypomethylation at *GFI1* in the peripheral blood of children suffering from sudden infant death syndrome (SIDS) compared with healthy children^35^, suggesting a complex triad relationship among intra utero tobacco smoke exposure, *GFI1* methylation status and SIDS.

In addition to the previously known CpG sites, we identified four novel CpG sites, including *ACSM3* cg0647882, *TRIM36* cg07469926, and cg05150608 in the intergenic region between *ANKRD9* and *RCOR1*, which represented a hypermethylated status in the Su-S group, and *SHANK2* cg05780228, representing a hypomethylated status in the Su-S group. NGS analysis revealed similar changes in DNA methylation of the adjacent CpG sites in *TRIM36* and *SHANK2*, suggesting that these sites are susceptible to maternal smoking. A recent meta-analysis suggested maternal smoking during pregnancy as a high-risk factor for neural tube defects^36^. *TRIM36* is expressed in the developing brain and is thought to be indispensable for somite formation^37^; lack of *TRIM36* due to a homozygous mutation has been reported to result in autosomal recessive anencephaly^38^. Recently, *TRIM36* hypermethylation has been linked to a cell-transformation effect of Benzo[a]pyrene, a polycyclic aromatic hydrocarbon found in tobacco smoke, in bronchial epithelial cells^39^. Hypermethylation status in *TRIM36* was also identified in tumours of lung, breast, liver and ovarian cancer^39^. Although no study has focused on *SHANK*2 hypomethylation, mutation and copy number variation of *SHANK2*, encoding a member of synaptic scaffolding protein in the SHANK family was reported in patients with autism spectrum disorder and mental retardation^40^. *ACSM3*, formerly known as an acyl-CoA synthetase medium chain family member 3, is associated with metabolic syndrome^41^. Recently, down-regulation of *ACSM3* has been implicated in the poor outcome of patients with hepatocellular carcinoma^42^. Like *SHANK2*, little is known about the epigenetic modification of *ACSM3*. The function of the CpG site, cg05150608, identified in the noncoding region between the *ANKRD9* and *RCOR1* genes is unknown. Taken together, our findings indicate changes in DNA methylation patterns of target genes, implicating prenatal tobacco smoke exposure as a risk factor for cancer, congenital abnormalities and neurodevelopmental disorders in the unborn child. However, our observations were limited to alterations in DNA methylation of cord blood, which may not represent methylation status in the foetal brain or other organs. Further studies are awaited to reinforce the relationship between tobacco smoke exposure and disease development as well as the role of novel target genes in this process.

Secondary comparison between the CpG sites identified in the Ne-S vs. Su-S comparison and those identified in the St-S vs. Su-S comparison pointed out *EVC2* cg01290904 as the only common CpG site, which was verified by NGS analysis, suggesting that this CpG site was affected by short-term tobacco smoke exposure in the first trimester. Mutation in *EVC2* causes congenital skeletal dysplasia, resulting in very short stature, known as Ellis-van Creveld syndrome^43^. Recent studies on *Evc2* mutant mice further implicate the importance of *EVC2* in the normal development of teeth and cranial bone^44,45^. Notably, children born to actively or passively smoking mothers show higher risk of hypodontia^46^. Moreover, a slightly increased risk of oral clefts has been reported in children born to actively smoking mothers^47^. These findings suggest a striking association between maternal smoking and *EVC2*. However, we could not find any previous study linking *EVC2* to early maternal smoking. Future studies focusing on *EVC2*-related outcomes and smoking exposure during the first trimester are needed to validate whether *EVC2* links maternal smoking with congenital anomalies in children.

The HM450K array allows access to >450,000 CpG sites across the genome that span 99% of the RefSeq genes and 96% of the CpG islands. Thus, HM450K array is a top choice for EWAS study. HM450K utilises the detection of DM CpG sites, providing insights into the affected genes. However, it is limited to the CpG sites included in the array and does not provide information on the neighbouring sites. Moreover, it is thought that the results of HM450K analysis may vary between different arrays (batch effect) or may not reflect bona fide DNA methylation due to problematic probes and intricacies in data normalisation and/or data analysis^23^. Therefore, with the availability of NGS in our laboratory, we chose to perform NGS analysis of DM CpG sites detected by HM450K. Using this approach helped us accomplish two objectives: to verify the changes in DNA methylation status of selected CpG sites identified using HM450K array and to assess the neighbouring CpG sites contained within the range of primers. The relatively strong correlation between results obtained with the two methods, together with the high-throughput capacity and single nucleotide resolution of NGS, favours the application of NGS for assessing DNA methylation of target gene(s) in large cohort studies. Moreover, with the increasing number of studies on DNA methylation as a biomarker for smoking^29,48^, especially maternal smoking, NGS may serve as a high-throughput and reliable approach for translational studies.

To the best of our knowledge, this is the first study to report the association of cord blood DNA methylation and prenatal cigarette smoking exposure in a Japanese population. Our study has several strengths. Firstly, all participants were native Japanese, living in Sapporo city and the surrounding areas, which were presumably homogenous in term of environment, culture, and daily life. Thus, the study population limited the effect of other environmental factors and was suitable for investigation of the interaction between a specific factor (prenatal smoking exposure) and the epigenome. Secondly, previously reported CpG sites were replicated in our studies, demonstrating the robustness of HM450K. Moreover, to overcome the drawbacks of HM450K, we applied bisulfite-NGS to validate our observations. Thirdly, by focusing on the windows of exposure with the exclusion of mothers who had stopped smoking before pregnancy, we demonstrated the effect of smoking on DNA methylation in the cord blood even if the mother stopped smoking as soon as the pregnancy was confirmed.

Although data were carefully analysed and verified, we are aware of the lack of a method of quantitative analysis of maternal smoking and the possible impact this may have on the results. In this study, mother–infant pairs were categorised based on self-reported information, without measuring any biomarkers for smoking (e.g. cotinine level). This approach might result in misclassification of maternal smoking groups. Nevertheless, the inaccuracy was presumed to be small, as a previous report on a Japanese population showed a high agreement between self-reported information and cotinine measurement^4^. Another limitation of this study might be that the effect of second-hand smoking was not accounted for in our analysis, which may lead to false unexposed status. However, this underestimation in smoking exposure would reduce the significance between Ne-S or St-S and Su-S groups. Thus, the CpG sites reported in this study are likely not overstated. Lastly, our results had shortcomings: replication in a second cohort and for the relatively small population studied, especially for Su-S and St-S groups (n = 46 and 77, respectively), which may reduce statistical strength and contribute to the absence of phenotypes, for instance, low birth weight, as discussed above. Therefore, the novel CpG sites should be interpreted with caution and must be validated in future cohort studies.

In conclusion, we reported novel CpG sites and verified previously established sites associated with maternal smoking and cessation of maternal smoking upon the confirmation of pregnancy. These sites were linked with adverse outcomes of intra utero tobacco smoke exposure, including recently reported effects on neurodevelopment disorders and congenital defects. However, the triad relation among maternal smoking, DNA methylation alterations and certain diseases remain unclear. To address this issue, future studies on the effects of maternal smoking on DNA methylation and disease development using large-scale cohorts are needed.

## Methods

### Study population

We performed a prospective study using Sapporo birth cohort from previously described Hokkaido Study on Environment and Children’s Health^49,50^. In this study, 514 women, who had enrolled at 23–35 weeks of gestation and delivered at the Sapporo Toho Hospital between 2002 and 2005 in Japan, agreed to participate. All participants were native Japanese and residents of Sapporo City or surrounding areas. Participants completed a questionnaire that asked for basic information, including their dietary habits, exposure to chemical compounds in their daily life, smoking history, alcohol consumption, caffeine intake, family income and educational levels of themselves and their partners. Of the 514 participants, 295 cord blood samples were obtained. Genomic DNA of sufficient quantity and quality was obtained from 292 participants.

### Genome-wide DNA methylation analysis

Umbilical cord blood samples were taken immediately after birth and stored at −80 °C. Genomic DNA was extracted from cord blood using a Maxwell^®^ 16 DNA Purification Kit (Promega, Madison, WI, USA). Genome-wide DNA methylation analysis was performed using the Infinium HumanMethylation 450 BeadChip (HM450K) (Illumina, San Diego, CA, USA) following the manufacturer’s instructions. All analyses were performed by G&G SCIENCE CO., LTD. (Matsukawa, Fukushima, Japan).

Methylation data were pre-processed using the R/Bioconductor package *minfi*^51^. From the 292 mother–infant pairs, one pair was rejected on the basis of poor quality control (QC) parameters. After QC, signal intensities were normalised using functional normalisation^52^. CpG probes containing single nucleotide pair-affected probes^53^, with a detection *P*-value of >0.05, sex chromosomes were removed^54^. As a result, 426,576 CpG probes were included in the working set. Inter-slide differences were adjusted using ComBat^55^. Methylation values of each CpG site were represented as β-values ranging from 0 to 1 and calculated from signal intensities prior to statistical analysis^56^.

### Data analysis

The 291 mother–infant pairs were categorised into three exposure classes based on the smoking history of mothers: 124 Ne-S, 46 Su-S, 77 St-S, who stopped smoking after the confirmation of their pregnancy; 44 St-S were excluded from this study because of the unknown duration of non-smoking periods. Robust linear regression analyses^57^ and empirical Bayesian methods^58^ were applied to determine the associations of β-value at each CpG site, adjusted for maternal age, infant sex, maternal education and surrogate variables. In addition, cell type fraction (CD4^+^ T cells, CD8^+^ T cells, granulocytes, monocytes, B cell and nucleated red blood cells) for each subject were calculated using reference-free cell mixture adjustments^59^. Statistical analyses were performed using *minfi*, *SVA, limma* and *RefFreeEWAS* packages in R ver. 3.2.3 and Bioconductor ver. 3.2. Differential methylated sites were identified between Ne-S and Su-S, Ne-S and St-S and St-S and Su-S. To correct for multiple testing, *P*-values were adjusted for FDR based on the Benjamini and Hochberg method to obtain *q*-values. Partial regression coefficient (β) for the magnitude of the effect on DNA methylation change and selected CpGs with an FDR of <0.05 and |β| >0.02. On specific genes, statistical significance of differences in methylation values between groups was determined using Steel–Dwass test; differences with *P*-value of <0.05 were considered statistically significant. Associations between DNA methylation ratios at the seven CpG sites obtained from HM450K analysis and from NGS analysis were tested using Spearman’s correlation coeffcients (ρ).

### Bisulfite next-generation sequencing

DNA was subjected to bisulfite conversion and amplified using FastStart Taq DNA Polymerase (Roche, Basel, Schweiz). PCR primers for bisulfite PCR were designed using MethPrimer (http://www.urogene.org/methprimer/) (Supplementary Table S5). For NGS, amplicon libraries were generated using an Ion Plus Fragment Library Kit (ThermoFisher Scientific, MA, USA) and Ion Xpress Barcode Adaptors Kit (ThermoFisher Scientific). Following Agencourt AMPure XP purification (Beckman Coulter, CA, USA), individual libraries were amplified using quantitative real-time PCR, diluted and pooled in equimolar ratios. The libraries were then processed with an Ion Chef System using an Ion PG Hi-Q Chef Kit (ThermoFisher Scientific). Sequencing was performed using an Ion PGM Hi-Q Sequencing Kit (ThermoFisher Scientific) and 850 flows on an Ion 318 Chip Kit v2 (ThermoFisher Scientific), according to the manufacturer’s protocol. After sequencing, single processing and base calling were performed using Torrent Suite 5.0.2 (ThermoFisher Scientific). Methylation analysis was performed using MethylationAnalysis_Amplicon plug-in v1.3 (ThermoFisher Scientific). Extreme outliers in methylation data can have significant influences on results. Therefore, outliers were removed using the Tukey method^60^. Comparison of methylation ratio among the different smoking groups was performed using Steel–Dwass test. Differences with *P*-value of <0.05 were considered statistically significant.

### Ethics

This study was conducted with informed consent of all subjects in writing. All procedures involving human subjects were approved by the University of Yamanashi, the Hokkaido University Graduate School of Medicine and the Hokkaido University Center for Environmental and Health Science and were performed in accordance with relevant guidelines and regulations.

### Data availability

The datasets generated and analysed during the current study are available from the corresponding author on request.

## Electronic supplementary material

Supplementary Figure and Tables
