# Supplementary material for: Association between DNA methylation in cord blood and maternal smoking: The Hokkaido Study on Environment and Children’s Health
Source: Sci Rep. 2018 Apr 4;8:5654. doi: 10.1038/s41598-018-23772-x (PMC5884848; doi:10.1038/s41598-018-23772-x)
Supplement: Supplementary file 1 — Supplementary Figure and Tables [file 41598_2018_23772_MOESM1_ESM.docx]

**Association between DNA methylation in cord blood and maternal smoking: The Hokkaido Study on Environment and Children’s Health**

Kunio Miyake^1,*^, Akio Kawaguchi^2,*^, Ryu Miura^3^, Sachiko Kobayashi^3^, Nguyen Quoc Vuong Tran^1^, Sumitaka Kobayashi^3^, Chihiro Miyashita^3^, Atsuko Araki^3^, Takeo Kubota^4^, Zentaro Yamagata^1^ & Reiko Kishi^3^

^1^Department of Health Sciences, Graduate School of Interdisciplinary Research, University of Yamanashi, Yamanashi, Japan

^2^Yamanashi Community Medicine Support Center, University of Yamanashi Hospital, Yamanashi, Japan

^3^Center for Environmental and Health Sciences, Hokkaido University, Hokkaido, Japan

^4^Faculty of Child Studies, Seitoku University, Iwase 550, Chiba, Japan

^*^Kunio Miyake and Akio Kawaguchi contributed equally to this work.

Correspondence and requests for materials should be addressed to K.M. (email: [kmiyake@yamanashi.ac.jp](mailto:kmiyake@yamanashi.ac.jp)).

**
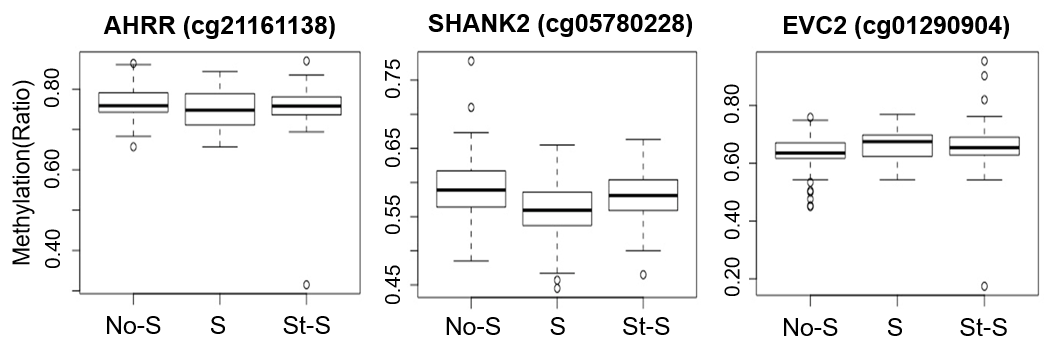
**

**Figure S1.** Box plot of next generation sequencing analysis of *AHRR* cg21161138, *SHANK2* cg05780228, and *EVC2* cg01290904 before removal of outliners, supplement to Figure 4 and Table 3. Black arrows indicate the outliers.

**
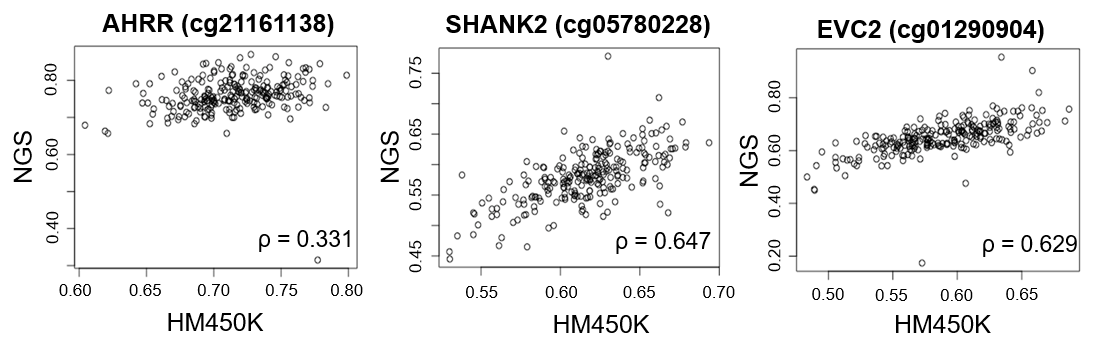
 Figure S2.** Correlation between HumanMethylation450K array (HM450K) and next generation sequencing (NGS) data analysis for the DNA methylation status of *AHRR* cg21161138, *SHANK2* cg05780228, and *EVC2* cg01290904 before removal of outliers, supplement to Figure 5. Values of Spearmen correlation coefficient (ρ) are showed. Black arrows indicate the outliers.

**
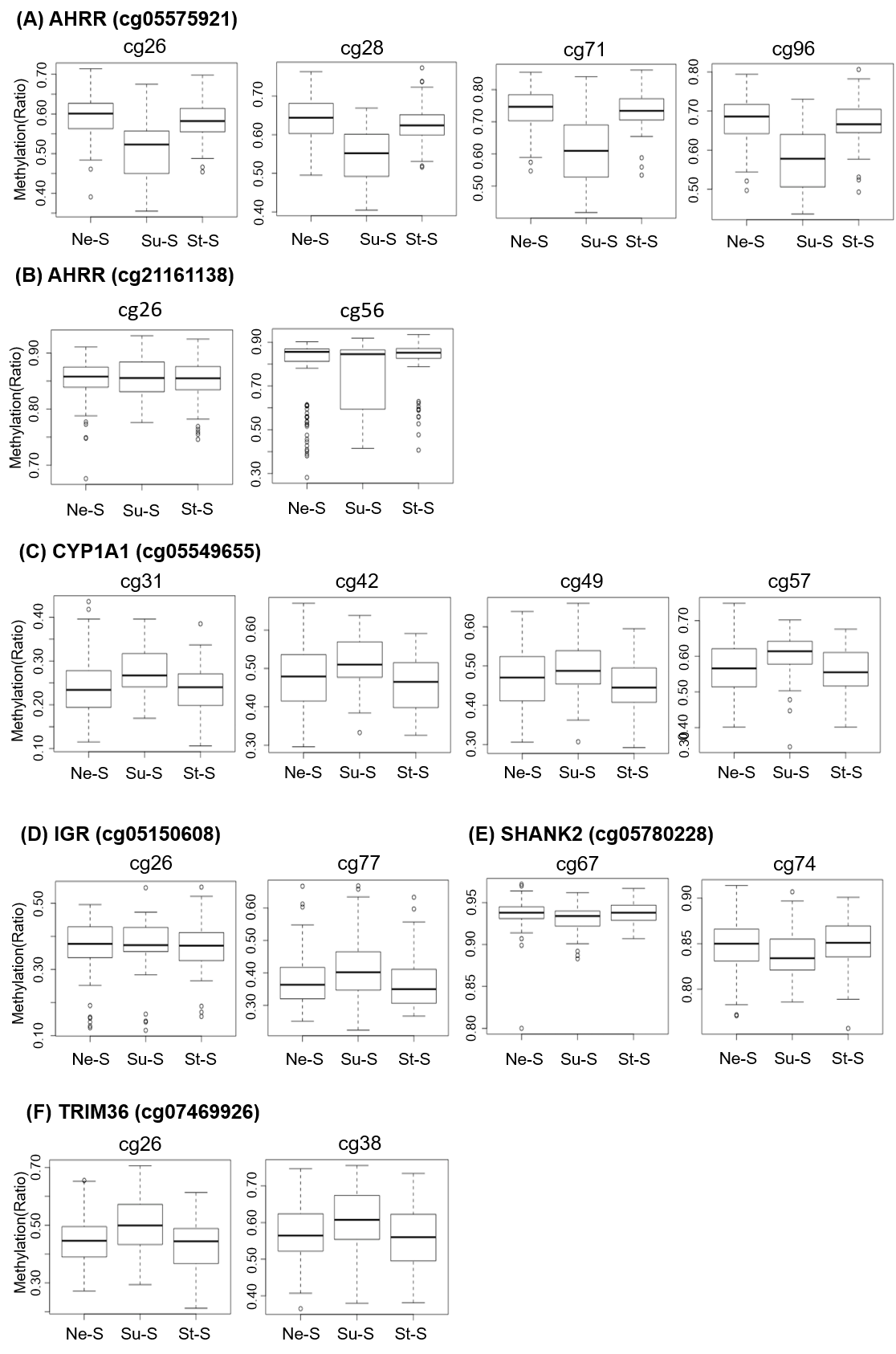
**

**Figure S**3. Box plots for DNA methylation ratio in other CpG sites covered by primers of next generation sequencing, supplement to Figure 4 and Table 3.

| **Table S1.** Differential methylation in cord blood DNA in the comparison between never-smokers (Ne-S) and sustained-smokers (Su-S). | | | | | | | | |
| --- | --- | --- | --- | --- | --- | --- | --- | --- |
| **CpG site** | **Chr.** | **Gene** | **Relation to gene** | **P-value** | | **FDR** | | **Beta** |
| cg05575921 | 5 | AHRR | Body | 9.65E-25 | | 4.12E-19 | | -0.083 |
| cg04063345 | 6 | ESR1 | Body | 7.18E-06 | | 0.035 | | -0.065 |
| cg12876356 | 1 | GFI1 | Body | 9.67E-08 | | 0.003 | | -0.063 |
| cg09935388 | 1 | GFI1 | Body | 4.82E-06 | | 0.029 | | -0.062 |
| cg18146737 | 1 | GFI1 | Body | 3.95E-07 | | 0.009 | | -0.057 |
| cg15626350* | 6 | ESR1 | Body | 1.29E-05 | | 0.048 | | -0.054 |
| cg09662411 | 1 | GFI1 | Body | 8.19E-07 | | 0.014 | | -0.035 |
| cg21161138* | 5 | AHRR | Body | 8.22E-12 | | 1.17E-06 | | -0.033 |
| cg25189904 | 1 | GNG12 | TSS1500 | 2.66E-07 | | 0.007 | | -0.033 |
| cg06338710 | 1 | GFI1 | Body | 4.93E-08 | | 0.002 | | -0.032 |
| cg20732787* | 19 | NCLN | Body | 1.16E-05 | | 0.046 | | -0.024 |
| cg05780228* | 11 | SHANK2 | Body | 8.23E-07 | | 0.014 | | -0.024 |
| cg11778756 | 11 | DDX10 | Body | 1.30E-08 | | 0.001 | | -0.023 |
| cg17710804 | 1 | C1orf113 | TSS1500;TSS200 | 1.25E-06 | | 0.016 | | -0.022 |
| cg18316974* | 1 | GFI1 | Body | 8.76E-06 | | 0.039 | | -0.022 |
| cg22242148* | 15 |  |  | 9.10E-07 | | 0.014 | | -0.022 |
| cg26098871* | 17 | ENGASE | 3'UTR | 2.86E-06 | | 0.022 | | -0.021 |
| cg09068004* | 5 | MARCH3 | TSS1500 | 2.34E-08 | | 0.001 | | 0.020 |
| cg09321403 | 6 |  |  | 3.49E-08 | | 0.002 | | 0.020 |
| cg11898431 | 6 |  |  | 1.11E-05 | | 0.045 | | 0.021 |
| cg15787636* | 17 | TBCD | Body | 5.31E-06 | | 0.030 | | 0.021 |
| cg04284530 | 5 | MARCH3 | TSS1500 | 1.89E-06 | | 0.020 | | 0.022 |
| cg12098750 | 12 | ANKS1B | TSS1500;Body | 2.82E-06 | | 0.022 | | 0.022 |
| cg06612130 | 4 |  |  | 1.49E-06 | | 0.016 | | 0.023 |
| cg01290904 | 4 | EVC2 | 5'UTR;Body | 5.90E-06 | | 0.031 | | 0.023 |
| cg17924476 | 5 | AHRR | Body | 1.85E-07 | | 0.006 | | 0.024 |
| cg13492331 | 10 | FAM107B | Body | 5.06E-06 | | 0.030 | | 0.026 |
| cg04333296* | 17 | LOC653653 | TSS200 | 5.44E-06 | | 0.030 | | 0.026 |
| cg23635789 | 20 | SRC | Body | 2.26E-06 | | 0.021 | | 0.026 |
| cg13932501 | 9 | AUH | Body | 7.71E-06 | | 0.036 | | 0.027 |
| cg07469926 | 5 | TRIM36 | Body | 3.64E-06 | | 0.025 | | 0.027 |
| cg15554505 | 19 |  |  | 9.54E-06 | | 0.041 | | 0.029 |
| cg04584103 | 14 | ASB2 | Body | 1.24E-06 | | 0.016 | | 0.029 |
| cg00394823 | 16 | ACSM3 | TSS200 | 5.12E-06 | | 0.030 | | 0.030 |
| cg05150608 | 14 |  |  | 7.49E-06 | | 0.036 | | 0.030 |
| cg05549655 | 15 | CYP1A1 | TSS1500 | 2.63E-07 | | 0.007 | | 0.031 |
| cg10078415 | 16 | ACSM3 | TSS1500 | 7.09E-06 | | 0.035 | | 0.035 |
| cg27434149 | 10 |  |  | 2.35E-06 | | 0.021 | | 0.043 |
| cg04180046 | 7 | MYO1G | Body | 2.41E-12 | | 5.13E-07 | | 0.044 |
| cg06478823* | 16 | ACSM3 | TSS1500 | 2.69E-07 | | 0.007 | | 0.048 |
| cg07340025 | 5 |  |  | 5.48E-06 | | 0.030 | | 0.049 |
| cg26493188 | 16 | FLYWCH1 | 5'UTR | 6.37E-06 | | 0.033 | | 0.054 |
| cg01215511 | 16 | FLYWCH1 | 5'UTR | 4.96E-06 | | 0.030 | | 0.062 |
| cg24228532 | 18 |  |  | 4.69E-06 | | 0.029 | | 0.063 |
| cg12803068 | 7 | MYO1G | Body | 7.46E-09 | | 0.001 | | 0.066 |
| cg22549041* | 15 | CYP1A1 | TSS1500 | 4.02E-06 | | 0.027 | | 0.070 |
| FDR < 0.05. \|β value\| > 0.02 | | |  |  |  | |  | |
| *Probes marked as low-quality by Naeem et al.^24^ which may contain multiple SNPs/INDELs or contain SNP at CpG site or have absolute beta difference between whole-genome bisulfite sequencing (WGBS) and Illumina HumanMethylation450 (HM450K) bead array is greater than 0.3 | | | | | | | | |

| **Table S2.** Differential methylation in cord blood DNA in the comparison between never-smokers (Ne-S) and stopped-smokers (St-S). | | | | | | |
| --- | --- | --- | --- | --- | --- | --- |
| **CpG site** | **Chr.** | **Gene** | **Relation to gene** | **P-value** | **FDR** | **Beta** |
| cg14418756 | 10 | STK32C | Body | 2.47E-06 | 0.031 | -0.058 |
| cg05618934* | 4 |  |  | 6.95E-07 | 0.019 | -0.042 |
| cg27173819 | 10 | STK32C | Body | 2.00E-06 | 0.031 | -0.041 |
| cg05225883 | 10 | STK32C | Body | 3.79E-06 | 0.036 | -0.035 |
| cg12594615 | 2 | TBC1D8 | Body | 4.50E-06 | 0.036 | -0.030 |
| cg24055029* | 6 | TNXB | Body | 1.79E-06 | 0.031 | -0.026 |
| cg04574346* | 12 |  |  | 2.25E-06 | 0.031 | -0.025 |
| cg19571540* | 14 |  |  | 2.12E-07 | 0.011 | -0.022 |
| cg03389650 | 7 |  |  | 4.54E-06 | 0.036 | -0.022 |
| cg24586758 | 5 | PCDHB3 | TSS200 | 2.22E-06 | 0.031 | -0.021 |
| cg02427511 | 16 |  |  | 3.83E-06 | 0.036 | 0.020 |
| cg01290904 | 4 | EVC2 | 5'UTR;Body | 2.52E-06 | 0.031 | 0.021 |
| cg17484472 | 16 |  |  | 5.43E-06 | 0.038 | 0.027 |
| cg27560452 | 16 |  |  | 8.60E-06 | 0.043 | 0.031 |
| cg26601609* | 8 |  |  | 6.32E-06 | 0.039 | 0.048 |
| FDR < 0.05. beta value > \|0.02\|  *Probes marked as low-quality by Naeem et al.^24^ which may contain multiple SNPs/INDELs or contain SNP at CpG site or have absolute beta difference between whole-genome bisulfite sequencing (WGBS) and Illumina HumanMethylation450 (HM450K) bead array is greater than 0.3 | | | | | | |

| **Table S3.** Differential methylation in cord blood DNA in the comparison between stopped-smokers (St-S) and sustained-smokers (Su-S). | | | | | | |
| --- | --- | --- | --- | --- | --- | --- |
| **CpG site** | **Chr.** | **Gene** | **Relation to gene** | **P-value** | **FDR** | **Beta** |
| cg05575921 | 5 | AHRR | Body | 5.81E-15 | 2.48E-09 | -0.073 |
| cg12876356 | 1 | GFI | Body | 2.03E-06 | 0.021 | -0.058 |
| cg06531741* | 11 | HTR3B | TSS200 | 2.34E-05 | 0.047 | -0.052 |
| cg06463313* | 21 | PFKL | Body | 2.33E-07 | 0.007 | -0.051 |
| cg09662411 | 1 | GFI1 | Body | 1.91E-05 | 0.044 | -0.034 |
| cg24733560* | 20 | TAF4 | Body | 3.76E-06 | 0.025 | -0.034 |
| cg21161138* | 5 | AHRR | Body | 9.50E-08 | 0.006 | -0.029 |
| cg14817490* | 5 | AHRR | Body | 5.30E-06 | 0.029 | -0.028 |
| cg06780726* | 13 | C13orf16 | Body | 2.11E-05 | 0.045 | -0.028 |
| cg09681977* | 2 | HDAC4 | Body | 1.99E-05 | 0.044 | -0.026 |
| cg20284239 | 1 | GPR161 | 5'UTR | 1.48E-05 | 0.039 | -0.024 |
| cg05780228* | 11 | SHANK2 | Body | 1.38E-05 | 0.038 | -0.022 |
| cg15813594 | 5 | EGFLAM | TSS200;Body | 6.81E-07 | 0.013 | -0.022 |
| cg21526530 | 15 | ATP8B4 | TSS1500 | 2.30E-05 | 0.047 | -0.021 |
| cg03923789 | 6 | PARK2 | Body | 1.02E-06 | 0.016 | -0.021 |
| cg23067299* | 5 | AHRR | Body | 1.28E-06 | 0.017 | 0.021 |
| cg09456465 | 1 |  |  | 1.90E-05 | 0.044 | 0.021 |
| cg12612445* | 21 | C21orf67 | Body | 5.25E-07 | 0.012 | 0.021 |
| cg10440011 | 17 | DPH1 | TSS1500 | 1.43E-05 | 0.039 | 0.021 |
| cg00213123* | 15 | CYP1A1 | TSS1500 | 3.87E-06 | 0.026 | 0.021 |
| cg12104266 | 20 | ZNF335 | TSS1500 | 1.11E-05 | 0.037 | 0.021 |
| cg00284551 | 15 | SNORD115-8 | TSS1500 | 8.31E-06 | 0.033 | 0.021 |
| cg10717610 | 2 |  |  | 9.99E-06 | 0.036 | 0.021 |
| cg06682024 | 2 | MERTK | Body | 1.51E-05 | 0.039 | 0.021 |
| cg21201401* | 20 | LIME1 | TSS200 | 6.72E-06 | 0.032 | 0.022 |
| cg17126924 | 17 |  |  | 7.21E-06 | 0.033 | 0.022 |
| cg13060531 | 1 | TTLL7 | TSS1500 | 2.78E-07 | 0.007 | 0.022 |
| cg24590430* | 10 |  |  | 6.95E-06 | 0.032 | 0.022 |
| cg10663316 | 12 |  |  | 2.06E-05 | 0.044 | 0.022 |
| cg16929104 | 19 | GDF15 | TSS200 | 5.62E-06 | 0.031 | 0.023 |
| cg18276112 | 7 | FOXK1 | Body | 1.28E-05 | 0.038 | 0.023 |
| cg23742314 | 12 |  |  | 4.74E-06 | 0.028 | 0.023 |
| cg19829353 | 6 | HECA | Body | 1.98E-05 | 0.044 | 0.023 |
| cg21809624 | 17 |  |  | 6.25E-06 | 0.032 | 0.024 |
| cg13630239 | 10 | RRP12 | 3'UTR | 7.37E-06 | 0.033 | 0.024 |
| cg11654024 | 1 |  |  | 9.00E-06 | 0.033 | 0.025 |
| cg02793451 | 16 | TOX3 | TSS1500 | 2.96E-06 | 0.023 | 0.026 |
| cg12537162 | 17 | ITGB3 | TSS1500 | 2.76E-06 | 0.022 | 0.026 |
| cg15345477 | 10 |  |  | 4.38E-06 | 0.028 | 0.026 |
| cg16449464 | 2 | MAP1D | TSS1500 | 1.07E-07 | 0.006 | 0.027 |
| cg12376422 | 9 |  |  | 2.48E-05 | 0.048 | 0.027 |
| cg03221914 | 6 | HIST1H2AJ | TSS1500 | 4.83E-06 | 0.029 | 0.027 |
| cg05549655 | 15 | CYP1A1 | TSS1500 | 9.95E-06 | 0.036 | 0.028 |
| cg05901543* | 16 | CDH15 | Body | 1.40E-05 | 0.039 | 0.029 |
| cg18107006 | 2 | HNRPLL | TSS1500 | 6.10E-07 | 0.012 | 0.029 |
| cg02459818* | 19 | VN1R4 | 1stExon | 2.48E-05 | 0.048 | 0.029 |
| cg01290565 | 12 | CUX2 | Body | 2.39E-05 | 0.048 | 0.029 |
| cg07469926 | 5 | TRIM36 | Body | 1.06E-06 | 0.016 | 0.029 |
| cg10817554* | 2 |  |  | 6.57E-06 | 0.032 | 0.029 |
| cg07807395 | 1 |  |  | 1.23E-06 | 0.016 | 0.030 |
| cg12738008* | 8 | MYOM2 | Body | 5.88E-06 | 0.032 | 0.030 |
| cg01624202 | 15 |  |  | 1.82E-05 | 0.043 | 0.030 |
| cg08720517 | 5 | LOC389333 | 1stExon | 7.71E-06 | 0.033 | 0.032 |
| cg25223623 | 5 |  |  | 1.90E-05 | 0.044 | 0.034 |
| cg05150608 | 14 |  |  | 2.56E-05 | 0.048 | 0.034 |
| cg06478823* | 16 | ACSM3 | TSS1500 | 2.40E-05 | 0.048 | 0.041 |
| cg16512885 | 1 |  |  | 3.11E-08 | 0.003 | 0.042 |
| cg22066599* | 11 |  |  | 6.05E-06 | 0.032 | 0.042 |
| cg23486701 | 2 | SPTBN1 | Body | 8.89E-06 | 0.033 | 0.042 |
| cg01590338 | 14 |  |  | 1.37E-05 | 0.038 | 0.044 |
| cg04717895 | 16 |  |  | 2.25E-06 | 0.021 | 0.045 |
| cg05225883 | 10 | STK32C | Body | 6.94E-06 | 0.032 | 0.046 |
| cg12101586 | 15 | CYP1A1 | TSS1500 | 1.25E-05 | 0.038 | 0.046 |
| cg08034867 | 17 | ABR | Body | 3.45E-06 | 0.024 | 0.053 |
| FDR < 0.05. beta value > \|0.02\|  *Probes marked as low-quality by Naeem et al.^24^ which may contain multiple SNPs/INDELs or contain SNP at CpG site or have absolute beta difference between whole-genome bisulfite sequencing (WGBS) and Illumina HumanMethylation450 (HM450K) bead array is greater than 0.3 | | | | | | |

| **Table S4.** Single-nucleotide polymorphisms that may affect result of HM450K | | | | | | |
| --- | --- | --- | --- | --- | --- | --- |
| **Gene** | **CpG site** | **SNPs** | **Distance to CpG site** | **Minor allele frequency** | **1000 Genomes Search** | |
|  |  |  |  |  | Japanese (in Tokyo, Japan) | Caucasian (British in England and Scotland; Finish in Finland; Utah Residents with North and Western European Ancestry) |
| CTCNAP2 | cg25949550 | rs111557555 | 21 | 0.0018 | G=1.0000; C=0.0000 | G=1.0000; C=0.0000 |
| ACSM3 | cg06478823 | None |  |  |  |  |
|  | cg10078415 | None |  |  |  |  |
|  | cg00394823 | None |  |  |  |  |
| FLYWCH1 | cg01215511 | None |  |  |  |  |
|  | cg26493188 | None |  |  |  |  |
| MARCH3 | cg04284530 | None |  |  |  |  |
|  | cg09068004 | None |  |  |  |  |
| IGR | cg05150608 | rs77154738 | 51 | 0.0018 | C=0.9856; T=0.0144 | C=1.0000; T=0.0000 |
| SHANK2 | cg05780228 | rs142581709 | 0 | 0.0009 | C=1.0000; T=0.0000 | C=1.0000; T=0.0000 |
|  |  | rs58493305 | 16 | 0.017811 | G=1.0000; A=0.0000 | G=1.0000; A=0.0000 |
|  |  | rs146007445 | 41 | 0.0005 | G=1.0000; A=0.0000 | G=1.0000; A=0.0000 |
| TRIM36 | cg07469926 | None |  |  |  |  |
| EVC2 | cg01290904 | rs78804781 | 26 | 0.005213 | C=1.0000; T=0.0000 | C=1.0000; T=0.0000 |

| **Table S5.** List of bisulphite PCR primers. | | | | |
| --- | --- | --- | --- | --- |
| **Name** | **Forward** | **Reverse** | **Length (bp)** | **Annealing (℃)** |
| ACSM3 (cg06478823) | TGTGGATTAGAGGATTTTGTGTTAG | CAATACTTCTTTTTCCACTCCAAATA | 91 | 59 |
| AHRR (cg05575921) | TATTTTTGAGAGGGTAGTTTTGTTT | AACCACTCCCAAAACCCAC | 120 | 59 |
| AHRR (cg21161138) | ATTTTAGTTTTAGGGTTTTTGAGGT | CTAATAATTAAAAAACCACCCCTA | 105 | 59 |
| CYP1A1 (cg05549655) | GTTATGTTAAATGGTATTGGGGTTT | AAAAAAAACAACCTACATATATCC | 81 | 59 |
| IGR (cg05150608) | TTGAATTTTTTTAAATTGGAGAAAT | CCTACACTAATTACAACTTCACCTAC | 103 | 57 |
| SHANK2 (cg05780228) | AGGTTGTGAAGGTATTTTTGGTTTA | CAACAAACAACTATATCCACTACTAAAAAA | 107 | 59 |
| TRIM36 (cg07469926) | AGAGGTGATGGTATAAAGGAGTATT | TAATAAAATATAAAACTACAAAATTTCAAA | 113 | 57 |
| EVC2 (cg01290904) | TTATTTTAGTTGTTGATGGGATTAG | AAACACACATAACCAAAACTCTTTC | 104 | 55 |
